# Effects of *rhBMP-2* gene transfection to periodontal ligament cells on osteogenesis

**DOI:** 10.1042/BSR20160585

**Published:** 2017-05-17

**Authors:** Cong-Xiang Jian, Quan-Shui Fan, Yong-He Hu, Yong He, Ming-Zhe Li, Wei-Yin Zheng, Yu Ren, Chen-Jun Li

**Affiliations:** 1 Department of Stomatology, PLA General Hospital of Chengdu Military Region, Chengdu 610083, China; 2 Chengdu Military Garrison Center for Disease Control and Prevention, Chengdu 650032, China

**Keywords:** Collagen type I, Gene transfection, Osteogenesis, Periodontal ligament cells, Recombinant human bone morphogenetic protein-2, Runx2

## Abstract

The present study aims to investigate the effect of recombinant human bone morphogenetic protein-2 (rhBMP-2) on the osteogenesis of periodontal ligament (PDL) cells. The expression vector of rhBMP-2 (pcDNA3.1-rhBMP-2) was established. PDL cells were obtained through the enzymatic digestion and tissue explant methods and verified by immunohistochemistry. Cells were classified into experimental (cells transfected with pcDNA3.1/rhBMP-2-EGFP), blank (cells with no transfection) and control group (cells transfected with empty plasmid). rhBMP-2 expression was assessed via Western blotting analysis. The mineralization ability, alkaline phosphatase (ALP) activity and level of related osteogenic biomarkers were detected to evaluate the osteogenic characteristics of PDL cells. The rhBMP-2 expression vector (pcDNA3.1-rhBMP-2) was successfully established. Primary PDL cells displayed a star or long, spindle shape. The cultured cells were long, spindle-shaped, had a plump cell body and homogeneous cytoplasm and the ellipse nucleus contained two or three nucleoli. Cells displayed a radial, sheaf-like or eddy-like arrangement after adherence growth. Immunohistochemical staining confirmed that cells originated from mesenchymal opposed to epithelium. The experimental group exhibited an enhanced mineralization ability, higher ALP activity and increased expression of rhBMP-2 and osteogenic biomarkers (Runx2, collagen type I and osteocalcin) than the blank and control group. The present study demonstrated that rhBMP-2 transfection enhances the osteogenesis of PDL cells and provides a possibility for the application of rhBMP-2 expression products in dental disease treatment.

## Introduction

The periodontal ligament (PDL) is the soft tissue that connects the cementum and alveolar bones. It plays an important role in supporting and maintaining teeth in the jaw bone, treating damaged tissue, maintaining homoeostasis and tooth nutrition [1]. The PDL comprises a diverse range of cells such as the cementoblasts (cells which promote the formation of cementum) and the osteoblasts (cells which facilitate bone formation). These cells have been proven to possess a series of characteristics such as the ability to develop mineralized nodules *in vitro* and express bone-associated markers alkaline phosphatase (ALP) and bone sialoprotein [2]. Human PDL stem cells (confirmed to be progenitor cells) were demonstrated to synthesize various tissues and share the features of other postnatal human mesenchymal stem cells (MSCs) such as osteogenic and chondrogenic differentiation capacities [3]. Therefore, the regenerative therapy has been widely adopted to reconstruct damaged periodontium as a result of periodontal disease [4].

Bone morphogenetic proteins (BMPs) belong to the transforming growth factor-β (TGF-β) superfamily and are a group of growing and secreted signalling proteins, which have a critical role in bone formation [5]. These proteins have been proven to be able to induce ectopic cartilage and bone formation by implanting into muscles. A previous study has demonstrated that BMP signals control the differentiation of osteoblasts, proliferation and differentiation of chondrocytes and bone quality [6]. Compared with other members of the BMPs family, BMP-2 is equipped with an especially strong osteoinductive function and is known as a growth factor in bone regeneration. It is capable of inducing the osteogenic differentiation of mesenchymal cells, *de novo* orthotopic or ectopic bone formation and accelerating the formation of new bone [7,8]. Furthermore, many other studies have demonstrated the successful healing of critical-sized mandible and calvarial defects through the use of osteoinductive BMPs [9,10]. Based on those clinical trials, the use of recombinant human BMP-2 (rhBMP-2) has become an FDA approved regenerative therapy for spinal fusion, alveolar ridge augmentation and sinus floor augmentation [11]. Jang et al. [12] found that rhBMP-2 boosts the osteogenesis of demineralized bone matrix in the mastoid obliteration model and is conducive to bone regeneration. Another study also displayed that MSCs transfected with the rhBMP-2 gene can increase osteogenic activity and increase the quantity of new bone formation [13]. Therefore, the present study was conducted to evaluate how transfecting the rhBMP-2 gene into PDL cells affects osteogenesis.

## Materials and methods

### Construction of pcDNA3.1-rhBMP-2 and pcDNA3.1/rhBMP-2-EGFP

The primer was designed according to the cDNA nucleotide sequence (NM001200) of rhBMP-2 (supplied by GenBank) with added BamHI and EcoRI restriction sites. The forward primer was (P1) 5′-TGGATCCTGACTCACGTCGGTCCTGT-3′ and the reverse primer was (P2) 5′-GCGACACCCACAACCCTCC–3′. PCR amplification was performed using cDNA obtained from osteosarcoma tissue as a template. Subsequently, the rhBMP-2 target gene fragment was connected with the plasmid vector through ligases and cloned into the pcDNA3.1vector. Therefore, the resulting product contained the plasmid of pcDNA3.1-rhBMP-2. This was determined by dual-enzyme digestion and sequencing.

The EGFP sequence was encoded according to the p-EGFP-C3 plasmid and the primer was designed by adding the BamHI and XbaI restriction sites. The forward primer was 5′-GCTAGGATCCCCGGTCGCCACCAT-3′ and the reverse primer was 5′-CCCTCTAGACCGTCGACTGCAGAATTCGAAG-3′. After purifying the PCR products, the targeted gene segment of rhBMP-2 and the EGFP fragment were digested using BamHI and EcoRI, BamHI and XbaI respectively. The purified fragment of pcDNA3.1 plasmid was digested using EcoRI and XbaI. The three fragments were connected using T4 ligase and the product was transformed into competent *Escherichia coli* cells. The colonies were selected based on if the LB culture medium contained ampicillin and then positive clones were picked. The plasmid was extracted using the plasmid isolation kit and then the pcDNA3.1/rhBMP-2-EGFP was obtained.

### Cell culture

A total of 32 teeth were extracted from six healthy patients aged between 15 and 30 years old. The teeth had no carious lesions, periapical periodontitis or periodontitis and were washed with PBS containing double-antibodies immediately after being separated from the oral cavity. They were then put into Dulbecco’s Modified Eagle’s Medium (DMEM) culture medium containing double-antibodies (penicillin and streptomycin), rinsed with double-resistant fluid and wetted with cell culture medium. One to three of the parodontiums were scraped from the root of the tooth with a scalpel. PDL cells were cultured via enzymic digestion in conjunction with the tissue explant method: PDL tissues were scraped from the teeth and immediately placed into a centrifuge tube, 5 ml of 0.2% collagenase type I was added followed by oscillation and digestion at 37°C for 110 min. Subsequently, the tissues were sufficiently dispersed and a small amount of DMEM culture medium (containing 10% FBS) was added to stop digestion. The tube was then centrifuged at 1000 rpm for 8 min, the supernatant was discarded and a small amount of DMEM culture medium (containing 10% FBS) was added. The tissues were inoculated into a 25 ml cell culture flask and uniformly disposed with a Pasteur pipette. After conversion, 8 ml of DMEM culture medium containing 20% FBS (Gibco Company, Grand Island, NY, U.S.A.) and double-antibodies were added to the cell culture flask and then placed in a CO_2_ thermostatic incubator (8% CO_2_, 37°C, humidity: 98%) for incubation. The cell culture flask was converted 4 h later and re-incubated until the cell growth covered 80% of the bottom of the flask. The initial culture medium was discarded, cells were washed with PBS and 0.25% trypsin was added for digestion for 5 min. Once most of the cells shrunk and become round upon observation under the inverted microscope, the digestion was stopped immediately with culture medium. Subsequently, the culture medium was centrifuged at 1000 rpm for 10 min and the supernatant was discarded. DMEM culture medium containing 10% FBS was added and the cells were passage cultured in 1:2 ratio.

### Identification of PDL cells

Identification by immunohistochemical staining occurred through the following process. The second generation of PDL cells were digested with 0.25% trypsin, inoculated on a glass slide at a concentration of 1 × 10^7^ cells/l, vibrated and then washed three times with PBS. Each wash lasted for 5 min. Subsequently, cells were fixed with 40 g/l of paraformaldehyde for 20 min, washed with PBS and then dried. H_2_O_2_ (3%) was added and after 10 min the normal serum working solution was added as a blocking agent for 10 min. After vimentin, cytokeratin (CK) and mouse anti-human antibodies (Invitrogen Inc., Carlsbad, CA, U.S.A.) were added, rabbit anti-mouse secondary antibody treatment and PBS washing occured (37°C, 0.5 h) and then the cells were stored overnight at 4°C. Diaminobenzidine (DAB) was applied for 5–6 min for developing and then the cells were counterstained with haematoxylin for 0.5–1 min. This was followed by upward gradient dehydration with ethanol, clearing with xylene and then sealing with gum. The cells were photographed under a microscope.

### Cell grouping

PDL cells were transfected with the expression vector (pcDNA3.1/rhBMP-2-EGFP) containing the rhBMP-2 gene according to the Lipofectamine2000™ transfection kit (Gibco, U.S.A.) specifications. This experiment contained three groups: a blank group (cells without gene transfection treatment), an experimental group (cells transfected with pcDNA3.1/rhBMP-2-EGFP) and a control group (cells transfected with empty plasmid). PDL cells (3 × 10^5^) were inoculated into each well of a six-well culture plate. After the cells were spread over a single layer, the culture medium was changed with an Opti-MEMI optimized cell culture medium. Transfection was performed 4 days later. A mixture of liposome and the rhBMP-2 gene was transfected in the experimental group. Opti-MEMI optimized cell culture medium was added to the control group in the same volume as the experimental group. Liposome (diluted with Opti-MEMI optimized cell culture medium) was also added to the blank group in the same volume as the experimental group. After mixing, the six-well culture plate was placed in a 5% CO_2_ incubator at 37°C for 4–6 h. Afterwards, the culture medium was changed with a normal complete culture medium and the six-well plate was placed back into the incubator for incubation. After 24 h of transfection, the previous medium was changed with a fresh culture medium. After 48 h, the expression of GFP in the blank, experimental and control groups were observed under a microscope to detect transfection efficiency.

### Western blotting

The cells from the three groups were digested with cell lysis buffer (0.1 mol/l NaCl, 0.01 mol/l Tris/HCl (pH 7.6), 0.001 mol/l EDTA (pH 8.0), 1 μg/l aprotinin and 100 μg/l phenylmethylsulphonyl fluoride) for 30 min and then centrifuged at 1200 rpm for 5 min to collect the supernatant. The amount of proteins were determined by the Coomassie Brilliant Blue method and an equal volume of sampling buffer was added. The resultant was boiled for 5 min and then sampled at 100 μg per strip. Subsequently, SDS/PAGE and Western blotting assay were performed. The Bio–Rad Gel Dol EZ imager (Bio–Rad Laboratories, Hercules, CA, U.S.A.) was used for developing and the ImageJ software was used to detect the grey value of the BMP-2 band. β-actin served as the internal reference. The concentration of spacer gel was 4.5% and the concentration of separation gel was 10%. The protein separated by electrophoresis was transformed into the PVDF membrane and 1:600 rhBMP-2 rabbit anti-human monoclonal antibody (Wuhan Boster Biological Technology Ltd., Wuhan, Hubei, China) was added into the blocked PVDF membrane. This was then stored overnight at 4°C. Subsequently, the protein was incubated with 1:1000 goat anti rabbit IgG (Wuhan Boster Biological Technology Ltd., Wuhan, Hubei, China), labelled with horseradish peroxidase for 3 h, washed and developed in DAB solution.

### MTT assay

When cell density of the three groups reached to 80%, the cells were washed twice with PBS and digested with 0.25% trypsin in preparation of single cell suspension. After counting, cells were inoculated into a 96-well plate at a density of 2 × 10^3^ cells/well. After culturing for 48 h, 20 μl of 5 mg/ml MTT solution (number A2776-1g, Shanghai Shifeng Biological Technology Co., Ltd., Shanghai, China) was added to the cells. Subsequently, the cells were incubated in an incubator for 4 h and the culture medium was discarded. DMSO (150 μl) was added to each well and then mixed for 10 min. At the 24, 48 and 72 h time points, the optical density (OD) value was measured at 490 nm using the ELISA reader. The cell viability curve was drawn with time as the *x*-axis and the OD value as the *y*-axis. The experiment was repeated three times.

### Alizarin Red staining

After cells of the three groups were taken out from the incubator, the culture media were discarded and mineralized culture media (α-MEN culture medium containing 8% FBS, 10^−7^ mol/l dexamethasone, 50 μg/ml ascorbic acid Vc and 10 mmol/l β-sodium glycerophosphate) were added for cell culture. When stratified cell growth and black nodules were observed, the cells were cultured for another 18 days and stained with Alizarin Red. The supernatant was discarded and the remaining solution was washed twice with PBS and then dried. Filtered 4% paraformaldehyde was added to fix the solution for 8 min and then the paraformaldehyde was removed. The remaining solution was washed with PBS, dried and added with Alizarin Red. The mixture was cultured in a CO_2_ incubator for 18 min, washed with PBS and then photographs were taken under a microscope. The area of the mineralized nodules was calculated using the image analysis software, Image-Pro Plus5.0 (Media Cybernetics, U.S.A.).

### Measurement of ALP activity

The cells of three groups were inoculated on to 96-well plates at a density of 5 × 10^4^ cells/ml. The examination was performed after cell lysis was observed under an inverted microscope (using the *p*-nitrobenzene phosphate azo method). The culture medium was discarded on the 0, 3rd, 7th and 14th days respectively. Cells were washed three times with PBS, dried and then rinsed with PBS three times. A reaction solution containing 25 mmol/l diethanol amine (buffer, EDA and AMP were purchased from Beijing Lidman Biochemical Technology Co., Ltd., China), 1 mmol/l magnesium chloride and 6.7 mmol/l PNPP in a 150 μl mixture was added into each well. These were then kept in the dark for 30 min at 37°C. Subsequently, 100 μl of 0.1 mol/l sodium hydroxide was added to stop the reaction. OD values were measured at a wavelength of 405 nm using a microplate reader (Biotek Corporation, U.S.A.) and the ALP standard curve enzyme activity values (U/l) were read.

### Quantitative real-time polymerase chain reaction

The cementum mRNA expression and osteogenesis markers were detected on the 3rd, 7th and 14th days. RNA of the transfected cells were extracted using TRIzol (Invitrogen, California, U.S.A.). The extracted RNA concentration was tested using the NanoDrop2000 (Thermo, Massachusetts, U.S.A.) and then stored at –80°C. The primers were designed using the Primer5.0 software and are shown in Table 1. The primers were all synthesized by Shanghai Invitrogen Biotechnology Co., Ltd. (Shanghai, China). The RNA samples were reversely transcribed into cDNA according to the reverse transcription kit specifications (TAKARA, article number: DRR047S). The cDNA obtained was diluted with 65 μl of diethyl phosphorocyanidate (DEPC) water, mixed and then the reaction system was prepared as 5 μl of SsoFast EvaGreen Supermix (Bio–Rad, article number: 1708882), 0.5 μl of forward primer (10 μM), 0.5 μl of reverse primer (10 μM) and 4 μl of cDNA. The PCR amplification conditions were: predegenerated at 95°C for 1 min, degenerated at 95°C for 30 s, annealing at 58°C for 5 s; for a total of 30 cycles and then extended at 72°C for 5 s. β-actin was used as the internal reference. Each gene from each sample was set at three repetitions. The expression of the osteogenesis markers Runx2, collagen type I, BMP-2 and osteocalcin were calculated using the 2^−ΔΔ*C*^_t_ method [14].

**Table 1:** The primer sequences for qRT-PCR

Gene	Forward primer	Reverse primer
Runx2	5′-CCCGTGGCCTTCAAGGT-3′	5′-CGTTACCCGCCATGACAGTA-3′
Collagen type I	5′-CCAGAAGAACTGGTACATCAGCAA-3′	5′-CGCCATACTCGAACTGGAATC-3′
Osteocalcin	5′-AGCAAAGGTGCAGCCTTTGT-3′	5′-GCGCCTGGGTCTCTTCACT-3′
BMP-2	5′-GGGCATCCTCTCCACAAA-3′	5′-GTCATTCCACCCCACGTC-3′
β-actin	5′-CCTGGCACCCAGCACAAT-3′	5′-GCTGATCCACATCTGCTGGAA-3′

qRT-PCR, quantitative real-time PCR.

### Statistical analysis

Data were statistically analysed using the SPSS 20.0 statistical software. Measurement data were presented as mean value ± S.D. ($\bar{\text{x}}$ ± s). The *t* test was utilized for the comparison between two groups and variance analysis was used for the comparison among multigroups. Enumeration data are presented as a percentage or ratio and the chi-squared test was used for comparison between groups. *P*<0.05 indicates a statistical significance.

## Results

### Identification of the recombinant plasmid

**Figure 1:**
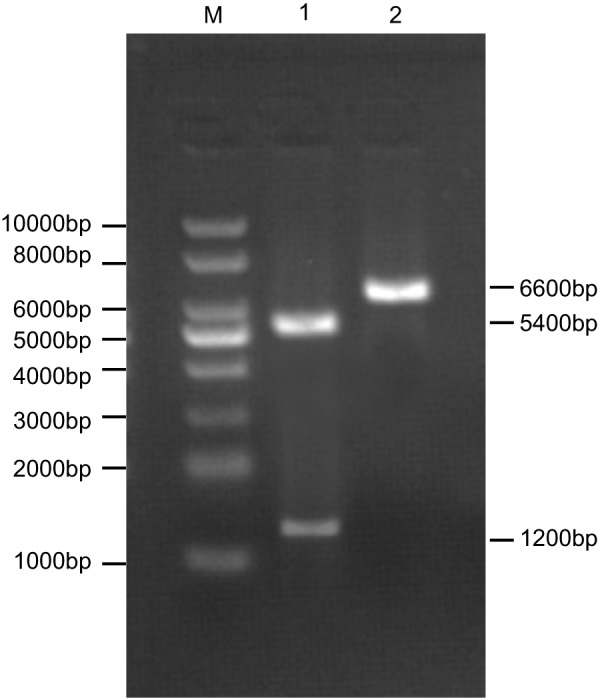
Electrophoresis for recombinant plasmid pcDNA3.1-rhBMP-2. M: DNA ladder marker with relative molecular weight 1 kbp; (1) enzyme-digested product 5.4 kbp (pcDNA3.1) and 1.2 kbp (target gene); (2) recombinant plasmid pcDNA3.1-rhBMP-2 (6.6 kbp).

As shown in Figure 1, the recombinant plasmid containing dual-enzyme digestion exhibited two bands at 1.2 and 5.4 kbp (1.2 kbp was rhBMP-2-cDNA product and 5.4 kbp was pcDNA3.1 linear plasmid). The recombinant plasmid without dual-enzyme digestion exhibited a band at 6.6 kbp (a recombinant plasmid pcDNA3.1-rhBMP-2). This indicates that the recombinant plasmid pcDNA3.1-rhBMP-2 was successfully constructed. The sequencing results of the recombinant plasmid was analysed using the BLAST software and was exactly the same as the GenBank code sequence (NM 001200) of *rhBMP-2*-mRNA in terms of homology. This further suggests that the plasmid was indeed the human BMP-2-cDNA fragment.

### Observation of cellular morphology and identification of cellular source

**Figure 2:**
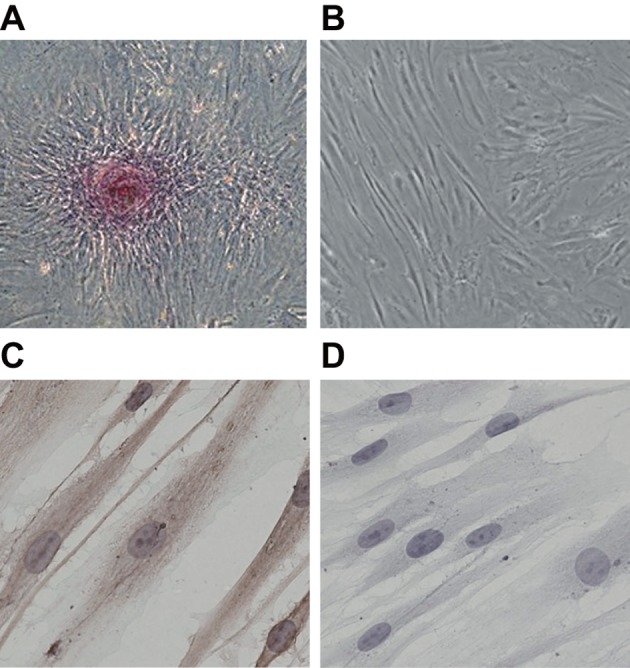
Microscope figures and immunohistochemical staining figures of PDL cells (**A**) primary PDL cells cultured by enzymic digestion in conjunction with tissue explant method; (**B**) passage cultured PDL cells; (**C**) anti-vimentin polyclonal antibody exhibited positive expression in cells; (**D**) anti-CK exhibited negative expression in cells.

Upon observation of PDL cells at primary culture under an inverted microscope, star or long spindle-shaped cells began to secrete from tissues on the 5th day (Figure 2A). Cells after passage culturing showed a long spindle shape and had a well-rounded cell body and homogeneous cytoplasm. The nuclei that displayed an oval shape were in the centre of the cell and had 2–3 nucleoli inside. The cells exhibited a radial, sheaf-like or eddy-like arrangement after cell adherence (Figure 2B).

The immunohistochemical staining results showed that the anti-vimentin polyclonal antibody (VIM) exhibited a positive expression (cytoplasm was brown) (Figure 2C). However, the anti-CK exhibited a negative expression (Figure 2D), suggesting that the cells are mesenchyme derived rather than epithelium derived.

### Efficiency of cell transfection

**Figure 3:**
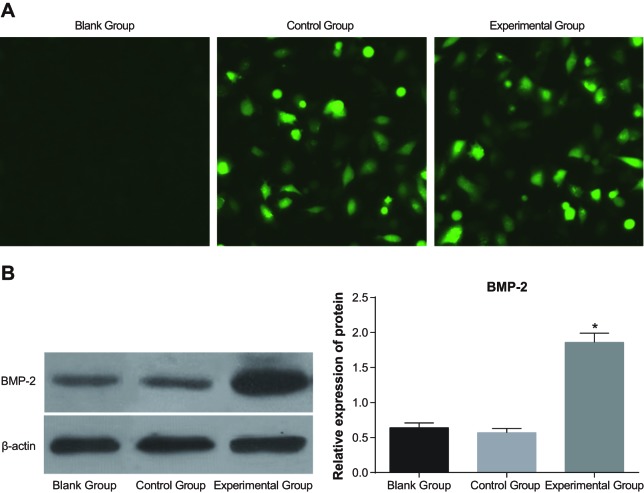
Efficiency and identification of cell transfection (**A**) efficiency of cell transfection under the fluorescence microscope; (**B**) band patterns of Western blotting. *, P,<0.05 compareds with the blank group.

After transfection for 48 h, strong green fluorescence was observed in the experimental group and the control groups, while no fluorescence was observed in the blank group under the microscope (×100) (Figure 3A). It was found by Western blotting that the bands of the blank and control groups were narrow and light in colour, while those of the experimental group were wide and dark in colour, suggesting that the BMP-2 expression of the experimental group was higher than that in the blank and control groups. It indicated that the expression of the transfected groups were increased significantly, and the *rhBMP-2* genes were successfully expressed (Figure 3B).

### Comparison of cell proliferative activity among the three groups

**Figure 4:**
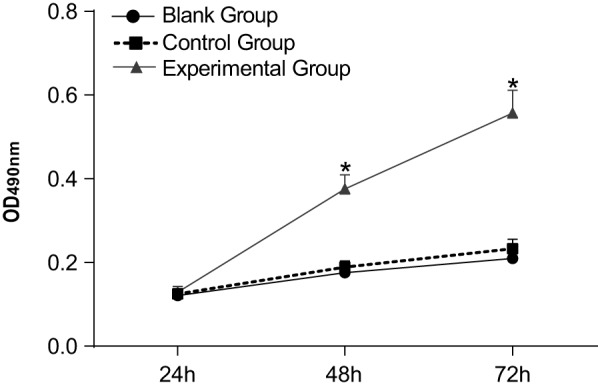
The curve of cell proliferative activity of three groups *P<0.05 compared with the control group and the blank group.

As presented in Figure 4, there was no significant difference in cell proliferative activity among the three groups at 24 h when the same initial concentration was kept (*P*<0.05). From 48 to 72 h, cell proliferative activity of the experimental group was higher than that of the blank group (*P*<0.05). There was no significant difference between the blank and control groups (*P*>0.05).

### Comparison of mineralization ability of PDL cells among the three groups

**Figure 5:**
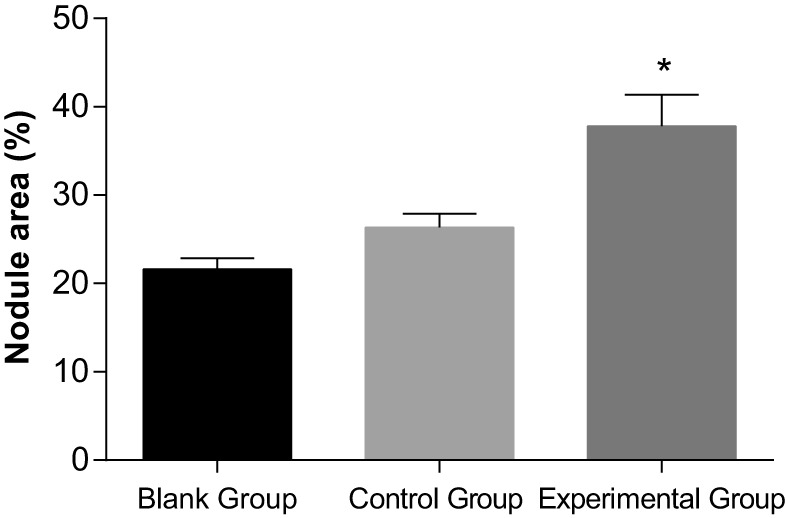
Comparison of Alizarin Red positive areas *P<0.05 compared with the control group and the blank group.

A larger Alizarin Red positive area suggests a stronger osteogenic capability. It was revealed that the positive areas of the PDL cells were significantly larger in the experimental group than that in the control and blank groups (*P*<0.05). There was no significant difference between the control and blank groups (*P*>0.05) (Figure 5). This suggests that the osteogenic capability of the experimental group was stronger than that of the control and blank groups.

### Comparison of ALP activity of PDL cells among the three groups

**Figure 6:**
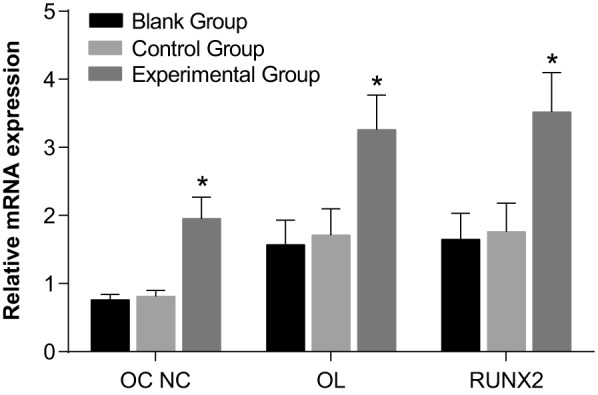
Comparisons of ALP activities of PDL cells among the blank group, the control group and the experimental group *P<0.05 compared with the blank group and the control group.

As shown in Figure 6, the ALP activity of cells in the three groups all increased with time. The ALP activity in the blank and control groups at the 0, 3rd, 7th and 14th days showed no significant difference (*P*>0.05). The ALP activity of the experimental group at the 3rd, 7th and 14th days were higher than those of the blank and control groups (*P*<0.05).

### Expressions of *osteocalcin, collagen type I, BMP-2* and *Runx2* mRNA of PDL cells in the three groups

**Figure 7:**
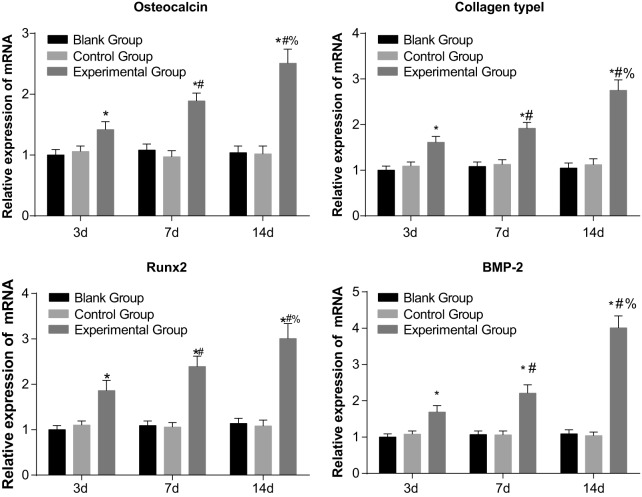
Comparisons of expressions of *osteocalcin, collagen type I, Runx2* and *BMP-2* mRNA of PDL cells among three groups *P<0.05 compared with the blank group and control group; ^#^*P*<0.05 compared with the 3rd day; ^%^*P*<0.05 compared with the 7th day.

The quantitative real-time PCR (qRT-PCR) results of the three osteogenesis markers are shown in Figure 7. The expressions of *osteocalcin, collagen type I*, Runx2 and *BMP-2* mRNA were not significantly different between the blank and control groups (*P*>0.05). However, the expressions of *osteocalcin, collagen type I, BMP-2* and *Runx2* mRNA of the PDL cells in the experimental group were significantly higher than those of the blank group (*P*<0.05). The expressions of *osteocalcin, collagen type I, BMP-2* and *Runx2* mRNA of the experimental group all increased over time (*P*<0.05).

## Discussion

PDL cells have been proven to be involved in many periodontal tissue processes such as normal metabolism, physiological health maintenance and reparative regeneration. It is also reported that the differentiation of PDL cell into osteoblast-like cells can be stimulated by mechanical stimuli [15]. A previous study indicated that the recombinant rhBMP-2 in an absorbable collagen sponge (ACS) carrier induces bone osseointegration and long-term functional loading of dental implants in dogs [16]. Furthermore, it is reported that the use of rhBMP-2 is able to promote osteogenesis and is becoming a clinical reality [17]. BMP-2 participates in bone formation, bone remodelling, bone development and osteoblast differentiation [18]. Therefore, the present study is designed to investigate the function of rhBMP-2 on the osteogenesis of PDL cells. Our data indicate that rhBMP-2 transfection enhances the osteogenesis of PDL cells and can promote the application of rhBMP-2 expression products in the treatment of dental diseases.

The initial findings of our study revealed that the experimental group exhibited higher expression of rhBMP-2 than the blank and control groups. This indicates that rhBMP-2 was successfully expressed in the experimental group. BMP-2 has demonstrated to be a significant and powerful osteo-inductive cytokine in clinical trials [19]. rhBMP-2 is a member of the TGF-β pleiotropic cytokines big family and can influence mitosis, chemotaxis and differentiation during osteogenesis [20]. rhBMP-2 has also been demonstrated to enhance osteoblast functioning in using various paths [7]. Hwang et al. [21] report that new bone formation in patients with bone defects is accelerated after the application of rhBMP-2 and maxillofacial cyst enucleation. It was also suggested by Haidar et al. [22] that the release of rhBMP-2 can be effectively extended through control over the release rate and as a result promote bone regeneration. The research from Chatzinikolaidou et al. [20] implied that bioactive rhBMP-2 release from the hybrid scaffolds can improve the control of osteogenic differentiation during cell culture. Li et al. [23] suggested that through the use of a carrier like fibrin, rhBMP-2 has a stronger enhancing effect on bone formation during distraction osteogenesis in rabbits. In addition, cell proliferative activity was highest in the experimental group. This suggests that TGF-β can exert a mediated effect on the proliferative regulation of PDL cells [24]. Fujii et al. [25] also demonstrated that exogenous TGF-β1 can stimulate the proliferation of human PDLs.

Furthermore, an enhanced mineralization ability and higher ALP activity was observed in the experimental group in comparison with the blank and control groups. ALP activity and mineralization ability are parameters applied to evaluate osteogenic differentiation [26,27]. In an *in vitro* study conducted by Lin et al. [28], a demineralized bone matrix was sequentially treated with heparin and rhBMP-2. The results showed that there was an increased calcium accumulation, higher ALP activity and more bone formation [28]. Guler et al. [29] found that BMP-2 biofunctionalized nanofibres are able to enhance *in vitro* osteogenic activity. He observed an enhanced calcium mineralization and higher ALP activity in cells after a 14-day *in vitro* culture of nanofibres with immobilized BMP-2 [29].

The data of our study displayed an increased expression of osteogenic biomarkers such as Runx2, collagen type I and osteocalcin in the experimental group. Runx2 acts as a transcription factor in the Runx family and Runx2 is a main target of the BMP pathway and is degraded by mediation of an ubiquitination pathway [30]. Furthermore, Runx2 is a specific marker gene involved in the bone formation process and is a major regulation factor in osteoblast differentiation [31,32]. Osteocalcin is a key marker for mature osteoblasts and collagen type I is the main organic agent of bone matrix and is a rudimentary product of the osteoblast differentiation process [18]. In addition, we also observed an elevated expression of osteopontin, osteocalcin and collagen type I and that the nuclear accumulation of Runx2 is increased when there is a higher osteogenetic differentiation of rat bone MSCs by BMP-2 [19].

To conclude, the present study demonstrated that rhBMP-2 transfection facilitates the osteogenesis of PDL cells by enhancing mineralization ability, increasing ALP activity as well as increasing the expression of the osteogenic biomarkers Runx2, collagen type I and osteocalcin. These results give us a better understanding of the osteogenesis of PDL cells. Therefore, our results provide promising possibilities for applying rhBMP-2 expression products in of the field of dentistry.

## Abbreviations

ALP: alkaline phosphatase
BMP: bone morphogenetic protein
CK: cytokeratin
DAB: diaminobenzidine
DMEM: Dulbecco’s Modified Eagle’s Medium
EGFP: enhanced green fluorescent protein
FDA: Food and Drug Administration
MSC: mesenchymal stem cell
OD: optical density
PDL: periodontal ligament
rhBMP-2: recombinant human bone morphogenetic protein-2
TGF-β: transforming growth factor-β

## References

[1] Shimono M., Ishikawa T., Ishikawa H., Matsuzaki H., Hashimoto S., Muramatsu T. (2003) Regulatory mechanisms of periodontal regeneration. Microsc. Res. Tech. 60, 491–502 12619125 10.1002/jemt.10290

[2] Seo B.M., Miura M., Gronthos S., Bartold P.M., Batouli S., Brahim J. (2004) Investigation of multipotent postnatal stem cells from human periodontal ligament. Lancet 364, 149–155 15246727 10.1016/S0140-6736(04)16627-0

[3] Park J.C., Kim J.M., Jung I.H., Kim J.C., Choi S.H., Cho K.S. (2011) Isolation and characterization of human periodontal ligament (PDL) stem cells (PDLSCs) from the inflamed PDL tissue: *in vitro* and *in vivo* evaluations. J. Clin. Periodontol. 38, 721–731 21449989 10.1111/j.1600-051X.2011.01716.x

[4] Zhang J., An Y., Gao L.N., Zhang Y.J., Jin Y. and Chen F.M. (2012) The effect of aging on the pluripotential capacity and regenerative potential of human periodontal ligament stem cells. Biomaterials 33, 6974–6986 22789721 10.1016/j.biomaterials.2012.06.032

[5] Davis H., Raja E., Miyazono K., Tsubakihara Y. and Moustakas A. (2016) Mechanisms of action of bone morphogenetic prote ins in cancer. Cytokine Growth Factor Rev. 27, 81–92 26678814 10.1016/j.cytogfr.2015.11.009

[6] Samara S., Dailiana Z., Varitimidis S., Chassanidis C., Koromila T., Malizos K.N. (2013) Bone morphogenetic proteins (BMPs) expression in the femoral heads of patients with avascular necrosis. Mol. Biol. Rep. 40, 4465–4472 23649763 10.1007/s11033-013-2538-y

[7] Kim S.E., Song S.H., Yun Y.P., Choi B.J., Kwon I.K., Bae M.S. (2011) The effect of immobilization of heparin and bone morphogenic protein-2 (BMP-2) to titanium surfaces on inflammation and osteoblast function. Biomaterials 32, 366–373 20880582 10.1016/j.biomaterials.2010.09.008

[8] Bae S.E., Choi J., Joung Y.K., Park K. and Han D.K. (2012) Controlled release of bone morphogenetic protein (BMP)-2 from nanocomplex incorporated on hydroxyapatite-formed titanium surface. J. Control Release 160, 676–684 22543042 10.1016/j.jconrel.2012.04.021

[9] Park J., Ries J., Gelse K., Kloss F., von der Mark K., Wiltfang J. (2003) Bone regeneration in critical size defects by cell-mediated BMP-2 gene transfer: a comparison of adenoviral vectors and liposomes. Gene Ther. 10, 1089–1098 12808439 10.1038/sj.gt.3301960

[10] Krebsbach P.H., Gu K., Franceschi R.T. and Rutherford R.B. (2000) Gene therapy-directed osteogenesis: BMP-7-transduced human fibroblasts form bone *in vivo*. Hum. Gene Ther. 11, 1201–1210 10834621 10.1089/10430340050015248

[11] Gao Q., Tong W., Luria J.S., Wang Z., Nussenbaum B. and Krebsbach P.H. (2010) Effects of bone morphogenetic protein-2 on proliferation and angiogenesis in oral squamous cell carcinoma. Int. J. Oral Maxillofac. Surg. 39, 266–271 20074910 10.1016/j.ijom.2009.11.015

[12] Jang Y.S., Choi C.H., Cho Y.B., Kang M.K. and Jang C.H. (2014) Recombinant human BMP-2 enhances osteogenesis of demineralized bone matrix in experimental mastoid obliteration. Acta Otolaryngol. 134, 785–790 24841764 10.3109/00016489.2014.900702

[13] Tsuda H., Wada T., Yamashita T. and Hamada H. (2005) Enhanced osteoinduction by mesenchymal stem cells transfected with a fiber-mutant adenoviral *BMP2* gene. J. Gene Med. 7, 1322–1334 15926193 10.1002/jgm.777

[14] Tuo Y.L., Li X.M. and Luo J. (2015) Long noncoding RNA UCA1 modulates breast cancer cell growth and apoptosis through decreasing tumor suppressive miR-143. Eur. Rev. Med. Pharmacol. Sci. 19, 3403–3411 26439035

[15] Liu M., Dai J., Lin Y., Yang L., Dong H., Li Y. (2012) Effect of the cyclic stretch on the expression of osteogenesis genes in human periodontal ligament cells. Gene 491, 187–193 22019432 10.1016/j.gene.2011.09.031

[16] Jovanovic S.A., Hunt D.R., Bernard G.W., Spiekermann H., Nishimura R., Wozney J.M. (2003) Long-term functional loading of dental implants in rhBMP-2 induced bone. A histologic study in the canine ridge augmentation model. Clin. Oral Implants Res. 14, 793–803 15015957 10.1046/j.0905-7161.2003.clr140617.x

[17] Wikesjo U.M., Sorensen R.G. and Wozney J.M. (2001) Augmentation of alveolar bone and dental implant osseointegration: clinical implications of studies with rhBMP-2. J. Bone Joint Surg. Am. 83-A, S136–S145 11314791

[18] Pyo S.J., Song W.W., Kim I.R., Park B.S., Kim C.H., Shin S.H. (2013) Low-level laser therapy induces the expressions of BMP-2, osteocalcin, and TGF-beta1 in hypoxic-cultured human osteoblasts. Lasers Med. Sci. 28, 543–550 22552925 10.1007/s10103-012-1109-0

[19] Sun J., Li J., Li C. and Yu Y. (2015) Role of bone morphogenetic protein-2 in osteogenic differentiation of mesenchymal stem cells. Mol. Med. Rep. 12, 4230–4237 26096280 10.3892/mmr.2015.3954 PMC4526091

[20] Chatzinikolaidou M., Pontikoglou C., Terzaki K., Kaliva M., Kalyva A., Papadaki E. (2016) Recombinant human bone morphogenetic protein 2 (rhBMP-2) immobilized on laser-fabricated 3D scaffolds enhance osteogenesis. Colloids Surf. B Biointerfaces 149, 233–242 27768913 10.1016/j.colsurfb.2016.10.027

[21] Hwang D.Y., On S.W. and Song S.I. (2016) Bone regenerative effect of recombinant human bone morphogenetic protein-2 after cyst enucleation. Maxillofac. Plast. Reconstr. Surg. 38, 22 27446821 10.1186/s40902-016-0070-4 PMC4937077

[22] Haidar Z.S., Hamdy R.C. and Tabrizian M. (2009) Delivery of recombinant bone morphogenetic proteins for bone regeneration and repair. Part A: current challenges in BMP delivery. Biotechnol. Lett. 31, 1817–1824 19690804 10.1007/s10529-009-0099-x

[23] Li Y., Li R., Hu J., Song D., Jiang X. and Zhu S. (2016) Recombinant human bone morphogenetic protein-2 suspended in fibrin glue enhances bone formation during distraction osteogenesis in rabbits. Arch. Med. Sci. 12, 494–501 27279839 10.5114/aoms.2016.59922 PMC4889683

[24] Ishibashi O., Ikegame M., Takizawa F., Yoshizawa T., Moksed M.A., Iizawa F. (2010) Endoglin is involved in BMP-2-induced osteogenic differentiation of periodontal ligament cells through a pathway independent of Smad-1/5/8 phosphorylation. J. Cell. Physiol. 222, 465–473 19918795 10.1002/jcp.21968

[25] Fujii S., Maeda H., Tomokiyo A., Monnouchi S., Hori K., Wada N. (2010) Effects of TGF-beta1 on the proliferation and differentiation of human periodontal ligament cells and a human periodontal ligament stem/progenitor cell line. Cell Tissue Res. 342, 233–242 20931341 10.1007/s00441-010-1037-x

[26] Wu T., Cheng N., Xu C., Sun W., Yu C. and Shi B. (2016) The effect of mesoporous bioglass on osteogenesis and adipogenesis of osteoporotic BMSCs. J. Biomed. Mater. Res. A 104, 3004–3014 27449696 10.1002/jbm.a.35841 PMC5995467

[27] Murgia A., Veronesi E., Candini O., Caselli A., D’souza N., Rasini V. (2016) Potency biomarker signature genes from multiparametric osteogenesis assays: will cGMP human bone marrow mesenchymal stromal cells make bone? PLoS ONE 11, e0163629 27711115 10.1371/journal.pone.0163629 PMC5053614

[28] Lin H., Zhao Y., Sun W., Chen B., Zhang J., Zhao W. (2008) The effect of crosslinking heparin to demineralized bone matrix on mechanical strength and specific binding to human bone morphogenetic protein-2. Biomaterials 29, 1189–1197 18083224 10.1016/j.biomaterials.2007.11.032

[29] Guler Z., Silva J.C. and Sarac A.S. (2016) Enhanced osteogenesis on biofunctionalized poly(varepsilon-caprolactone)/poly(m-anthranilic acid) nanofibers. J. Biomater. Appl. 31, 743–754 27440863 10.1177/0885328216660379

[30] Dong M., Jiao G., Liu H., Wu W., Li S., Wang Q. (2016) Biological silicon stimulates collagen type 1 and osteocalcin synthesis in human osteoblast-like cells through the BMP-2/Smad/RUNX2 signaling pathway. Biol. Trace Elem. Res. 173, 306–315 27025722 10.1007/s12011-016-0686-3

[31] Matsubara T., Kida K., Yamaguchi A., Hata K., Ichida F., Meguro H. (2008) BMP2 regulates Osterix through Msx2 and Runx2 during osteoblast differentiation. J. Biol. Chem. 283, 29119–29125 18703512 10.1074/jbc.M801774200 PMC2662012

[32] Wu M., Hesse E., Morvan F., Zhang J.P., Correa D., Rowe G.C. (2009) Zfp521 antagonizes Runx2, delays osteoblast differentiation *in vitro*, and promotes bone formation *in vivo*. Bone 44, 528–536 19095088 10.1016/j.bone.2008.11.011 PMC2746087

